# MMP-12, Secreted by Pro-Inflammatory Macrophages, Targets Endoglin in Human Macrophages and Endothelial Cells

**DOI:** 10.3390/ijms20123107

**Published:** 2019-06-25

**Authors:** Mikel Aristorena, Eunate Gallardo-Vara, Matej Vicen, Mateo de Las Casas-Engel, Luisa Ojeda-Fernandez, Concepción Nieto, Francisco J. Blanco, Ana C. Valbuena-Diez, Luisa M. Botella, Petr Nachtigal, Angel L. Corbi, María Colmenares, Carmelo Bernabeu

**Affiliations:** 1 Centro de Investigaciones Biológicas, Consejo Superior de Investigaciones Científicas (CSIC), 28040 Madrid, Spain; m.aristorena@ucl.ac.uk (M.A.); eunate.gallardo@yale.edu (E.G.-V.); mcasas@cib.csic.es (M.d.L.C.-E.); mluisa.ojeda@gmail.com (L.O.-F.); cnieto@cib.csic.es (C.N.); fjblanco@ugr.es (F.J.B.); ac22vd@hotmail.com (A.C.V.-D.); cibluisa@cib.csic.es (L.M.B.); acorbi@cib.csic.es (A.L.C.); maria.colmenares@cib.csic.es (M.C.); 2 Centro de Investigación Biomédica en Red de Enfermedades Raras (CIBERER), 28040 Madrid, Spain; 3 Department of Biological and Medical Sciences, Faculty of Pharmacy in Hradec Kralove, Charles University, 500 05 Hradec Kralove, Czech Republic; matej.vicen@gmail.com (M.V.); nachtigal@faf.cuni.cz (P.N.)

**Keywords:** monocytes, macrophages, endothelial cells, inflammation, MMP-12, endoglin

## Abstract

Upon inflammation, monocyte-derived macrophages (MΦ) infiltrate blood vessels to regulate several processes involved in vascular pathophysiology. However, little is known about the mediators involved. Macrophage polarization is crucial for a fast and efficient initial response (GM-MΦ) and a good resolution (M-MΦ) of the inflammatory process. The functional activity of polarized MΦ is exerted mainly through their secretome, which can target other cell types, including endothelial cells. Endoglin (CD105) is a cell surface receptor expressed by endothelial cells and MΦ that is markedly upregulated in inflammation and critically involved in angiogenesis. In addition, a soluble form of endoglin with anti-angiogenic activity has been described in inflammation-associated pathologies. The aim of this work was to identify components of the MΦ secretome involved in the shedding of soluble endoglin. We find that the GM-MΦ secretome contains metalloprotease 12 (MMP-12), a GM-MΦ specific marker that may account for the anti-angiogenic activity of the GM-MΦ secretome. Cell surface endoglin is present in both GM-MΦ and M-MΦ, but soluble endoglin is only detected in GM-MΦ culture supernatants. Moreover, MMP-12 is responsible for the shedding of soluble endoglin in vitro and in vivo by targeting membrane-bound endoglin in both MΦ and endothelial cells. These data demonstrate a direct correlation between GM-MΦ polarization, MMP-12, and soluble endoglin expression and function. By targeting endothelial cells, MMP-12 may represent a novel mediator involved in vascular homeostasis.

## 1. Introduction

Inflammation is an adaptive response that is triggered by noxious stimuli and conditions, such as infection and tissue injury [1]. Macrophages (MΦ) are critically involved in host defense by regulating inflammatory responses, as well as tissue remodeling, repair, and healing [2]. In inflammation-related cardiovascular disease, MΦ represent a major component of vessel wall infiltrates where they display great plasticity regulating a variety of pathophysiological processes [3,4], including atherosclerosis, myocardial infarction, atrial fibrilation, stroke, homeostatic arterial remodeling, hypertension, angiogenesis, or vasculitides [5,6,7,8,9]. It is widely accepted that the recruitment of MΦ to vascular inflammatory lesions involve the transit of circulatory monocytes through the vascular endothelium, a process associated with their activation/differentiation to MΦ [5,10,11]. This migration of monocytes involves adhesive interaction of their cell surface integrins with receptors expressed on endothelial cells [12,13,14]. Once differentiated, MΦ release inflammatory mediators that regulate vascular function in a process that is poorly understood.

An adequate regulation of macrophage polarization is crucial for a fast and efficient initial response (pro-inflammatory GM-MΦ) and a good resolution (anti-inflammatory M-MΦ) of the inflammatory process. Human MΦ are classified according to the activation stimuli into, at least, two polarization states based on their profiles of secreted proteins, including cytokines and matrix metalloproteinases (MMPs) [15]. Thus, MΦ generated in the presence of GM-CSF or M-CSF are representative of the pro-inflammatory (GM-MΦ) or the anti-inflammatory (M-MΦ) polarization states, respectively [16,17]. Among MΦ-secreted proteins, MMPs are a family of proteases that contain a zinc atom at their active site and are able to regulate endothelial sprouting and angiogenesis by degrading different extracellular matrix proteins, including collagen, laminin, and elastin [18,19]. Human elastase, also known as MMP-12, was first identified as an elastolytic metalloproteinase secreted by inflammatory alveolar MΦ [20]. MMP-12 is considered not only as a GM-MΦ pro-inflammatory marker [21,22] but also as a pro-inflammatory protease whose expression is induced by the cytokine GM-CSF [23,24]. The presence of MMP-12 has been reported in inflammation-associated atherosclerosis [25,26] and other vascular pathologies like aortic dissection, retinopathy, intracerebral hemorrhage, peripheral vascular damage, arterial stiffening, obstructive pulmonary disease (COPD), or deep vein thrombosis [27]. Moreover, the atherosclerotic plaque MMP-12-positive MΦ subset predicts adverse outcomes after endarterectomy [28], while MMP-12 inhibition retards atherosclerotic plaque development in a mouse model [29]. Furthermore, MMP-12 inhibits wound healing and endothelial-driven angiogenesis in vitro and in vivo while promoting endothelial cell apoptosis [30,31,32,33,34]. In spite of the functional effects of MMP-12 on the vascular endothelia, many of its target endothelial proteins remain to be identified.

Endoglin (CD105) is a membrane glycoprotein that is highly expressed in endothelial cells [35] and at lower levels in MΦ [36,37]. It is an auxiliary receptor for the members of the transforming growth factor-β (TGF-β) family, and a role for endothelial endoglin in pathological conditions involving the vasculature, including hereditary hemorrhagic telangiectasia type 1 (HHT1), preeclampsia, and cancer angiogenesis, has been described [38,39,40,41]. Endoglin expression in endothelia is strongly upregulated and is consistently associated with an infiltrate of inflammatory cells during inflammation and dermal wound healing in vivo [42]. In this context, a regulatory role for endoglin in transendothelial monocyte trafficking has been suggested, by which endothelial endoglin interacts with monocyte integrins, and this cell-cell adhesion process is stimulated by inflammatory stimuli [13]. In addition to the membrane-bound protein, high levels of circulating endoglin (soluble endoglin; sEng) in plasma from patients with preeclampsia, cancer, or inflammatory-related diseases such as atherosclerosis, psoriasis, or rheumatoid arthritis, has been reported [40,41,43,44,45,46,47]. Shedding of the ectodomain of endoglin can be triggered by inflammation, tumor necrosis factor α (TNF-α), endothelial injury, or anti-endoglin antibodies [47,48,49,50,51]. Of note, sEng (i) displays pro-inflammatory activity via nuclear factor-kappa B (NFκB) and interleukin 6 (IL6) in human endothelial cells [52]; (ii) shows antiangiogenic activity and increased vascular permeability in vitro and in vivo [40,51,53]; (iii) modulates inflammation-associated monocyte adhesion and transmigration [13]; (iv) contributes to endothelial dysfunction, as shown in transgenic animals overexpressing human sEng [54,55]; and (v) regulates vascular development and arteriovenous malformations by modulating angiogenesis [47]. Therefore, identification of the mechanisms responsible for the generation of sEng in the inflammatory context may provide novel therapeutic targets for inflammation-related vascular pathologies. Here, we provide evidence that the metalloelastase MMP-12 is the major MΦ protease that targets membrane-bound endoglin in MΦ and endothelial cells, leading to the shedding of soluble endoglin in inflammatory associated processes, and so the consideration of both MMP-12 and soluble endoglin as correlated GM-MΦ polarization markers. This is the first time that the human GM-MΦ/MMP-12/soluble endoglin axis is functionally reported in an inflammatory context.

## 2. Results

### 2.1. Inhibitory Effect of the GM-MΦ Secretome on Endothelial Tubulogenesis

**Figure 1:**
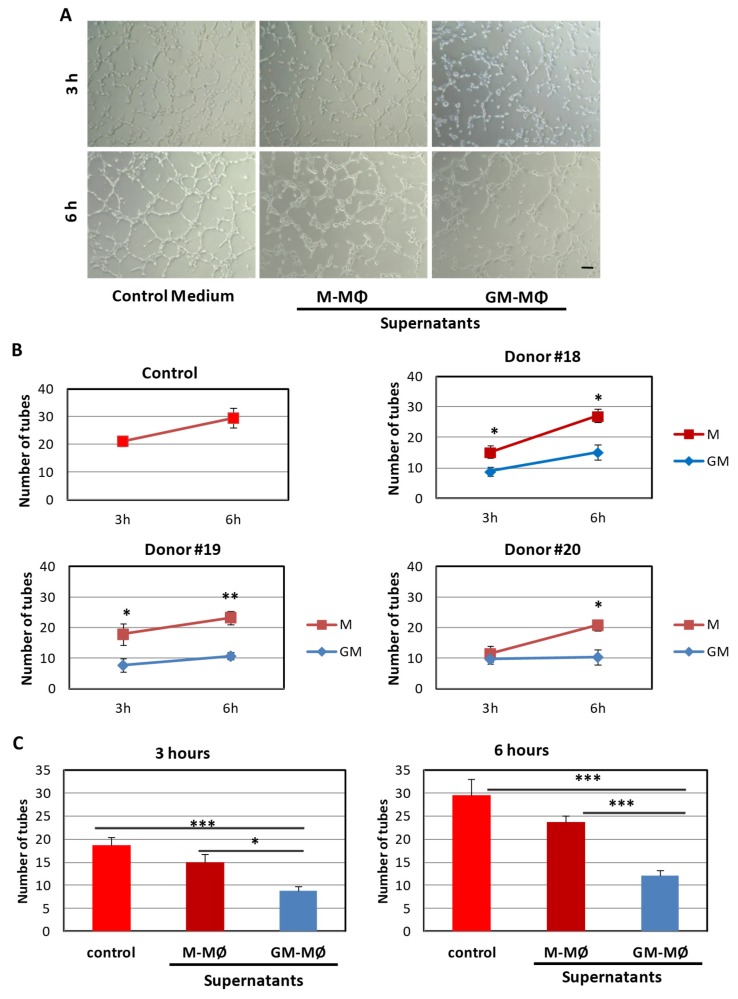
Effect of GM-MΦ and M-MΦ supernatants on tubulogenesis. Matrigel was mixed (at 1:1 dilution) with control medium or macrophage (MΦ) supernatants from three different donors (#18, #19, and #20), and the mixture was added to plates and incubated for 1 h. Then, human umbilical vein-derived endothelial cells (HUVECs) in vascular endothelial growth factor (VEGF)-enriched EBM2/EGM2 medium were added to Matrigel plates and incubated for the times indicated at 37 °C. The cord network formation was visualized by taking pictures at 3 h and 6 h after cell plating and representative images are shown (**A**). The appearance of an almost complete network is achieved by 6 h in cells incubated with control medium or cells treated with M-MΦ supernatants; while in the presence of GM-MΦ supernatants, open tubules with some patches of disorganized and sparse cells are observed. Scale bar: 100 μm. The number of closed tubes in the network was quantified and representative assays of more than three different experiments per condition are shown (**B**). The mean number of closed tubes, representing all the experiments (*n* = 6), is shown by histograms (**C**). Statistical significance was calculated with one way ANOVA and data are presented as mean ± SEM. (*p < 0.05; **p < 0.01; ***p < 0.001).

Human monocytes were cultured for six days in the presence of GM-CSF (GM-MΦ) or M-CSF (M-MΦ), and culture supernatants were collected. Then, the effect of these supernatants on endothelial tubulogenesis, a crucial step in the formation of functional blood vessels during angiogenesis, was tested. As shown in Figure 1, culture supernatants from GM-MΦ, but not from M-MΦ, significantly decreased endothelial tubulogenesis at two different times points (3 h and 6 h), and this effect was confirmed with MΦ derived from at least five different donors. In addition, wound healing experiments (*n* = 4) in human endothelial monolayers showed that culture supernatants from GM-MΦ (27.21% +/− 1.28 wound closure vs. 42.84% +/− 12.4 of control; *p* = 0.048), but not from M-MΦ (44.57% +/− 4.70; no significant difference vs. control), significantly decreased endothelial cell migration at 8 h post-wound. These results suggest that the GM-MΦ secretome exerts an anti-angiogenic activity on endothelial cells.

**Figure 2:**
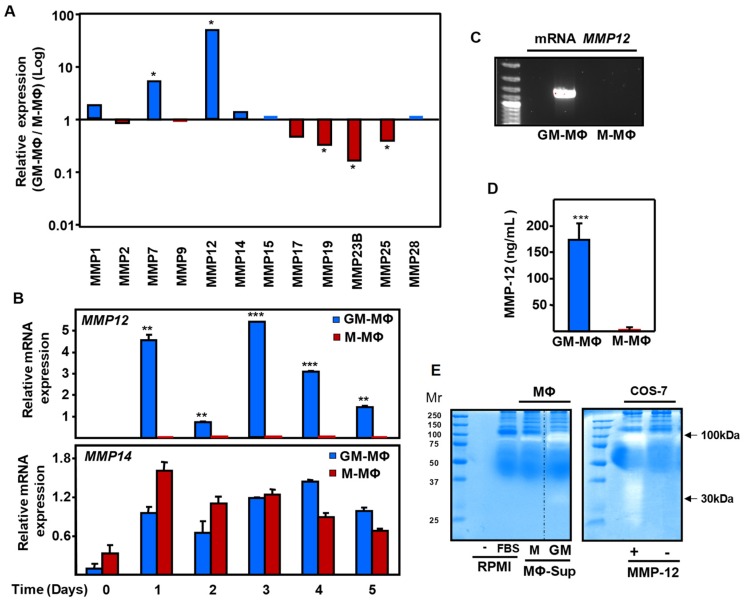
Expression profile of matrix metalloproteinases (MMPs) in GM-MΦ and M-MΦ. Human monocytes were incubated with GM-CSF or M-CSF, resulting in GM-MΦ and M-MΦ, respectively. (**A**) The relative mRNA expression levels of different MMPs was determined by qRT-PCR and represented by the GM-MΦ/M-MΦ ratio. (**B**) mRNA expression levels of MMP-12 and MMP-14 along GM-MΦ and M-MΦ polarization, as determined by qRT-PCR. The statistical significance of GM-MΦ versus M-MΦ data is shown. (**C**) MMP-12 mRNA expression of GM-MΦ and M-MΦ analyzed by RT-PCR, followed by agarose gel analysis of the product. (**D**) MMP-12 protein levels in culture supernatants at the final stage of differentiation were measured by ELISA. (**E**) Caseinolytic zymography assay of culture supernatants of GM-MΦ and M-MΦ (left panel), and COS-7 cells transfected with or without an expression vector encoding MMP-12 (right panel). A control of RMPI medium with or without fetal bovine serum (FBS) was included in the left panel. The caseinolytic activity was revealed by the white staining over the blue background of total proteins stained with Coomassie Brilliant Blue. Statistical analysis of triplicates was calculated using the paired two-tailed Student *t* test and data are presented as mean ± SD. (*p < 0.05; **p < 0.01; ***p < 0.001).

In order to identify the specific component of the GM-MΦ secretome involved in this inhibitory activity, we next analyzed the relative expression profile of different MMPs in GM-MΦ and M-MΦ by qRT-PCR. Of note, the family of MMPs has been shown to regulate vascular endothelial function [18,19]. As shown in Figure 2A, the metalloelastase MMP-12 displayed the highest GM-MΦ/M-MΦ ratio among 12 different MMPs analyzed. In addition, most of the MMPs, except MMP-7, showed relatively low expression levels in GM-MΦ compared to M-MΦ. A kinetic study showed a marked differential expression of the MΦ MMP-12 mRNA, as it was expressed at much higher levels in pro-inflammatory GM-MΦ than in M-MΦ (Figure 2B,C), which is in line with a previous report [21]. This differential expression of MMP-12 was seen at all time points during MΦ differentiation, which is in agreement with the reported effects of GM-CSF on GM-MΦ [23,24]. As a control, the kinetic expression levels of MMP-14 showed similar expression levels in GM-MΦ and M-MΦ during all time points (Figure 2B). The differential upregulated expression of MMP-12 in GM-MΦ versus M-MΦ was also demonstrated at the protein level by ELISA (Figure 2D), and its biological significance was corroborated by a zymography assay on casein. As shown in Figure 2E, GM-MΦ supernatants exhibited a higher MMP-12-specific peptidase activity as compared to M-MΦ supernatants. As a control, the activity of the secreted MMP-12 from transfected COS-7 cells was also evident (Figure 2E). In fact, the digestion pattern observed in MMP-12 transfectants was similar to that obtained using GM-MΦ supernatants, with the appearance of two specific bands around 100- and 30-kDa. Conversely, no specific digestion bands were observed in supernatants from either M-MΦ or mock-transfected COS-7 cells.

**Figure 3:**
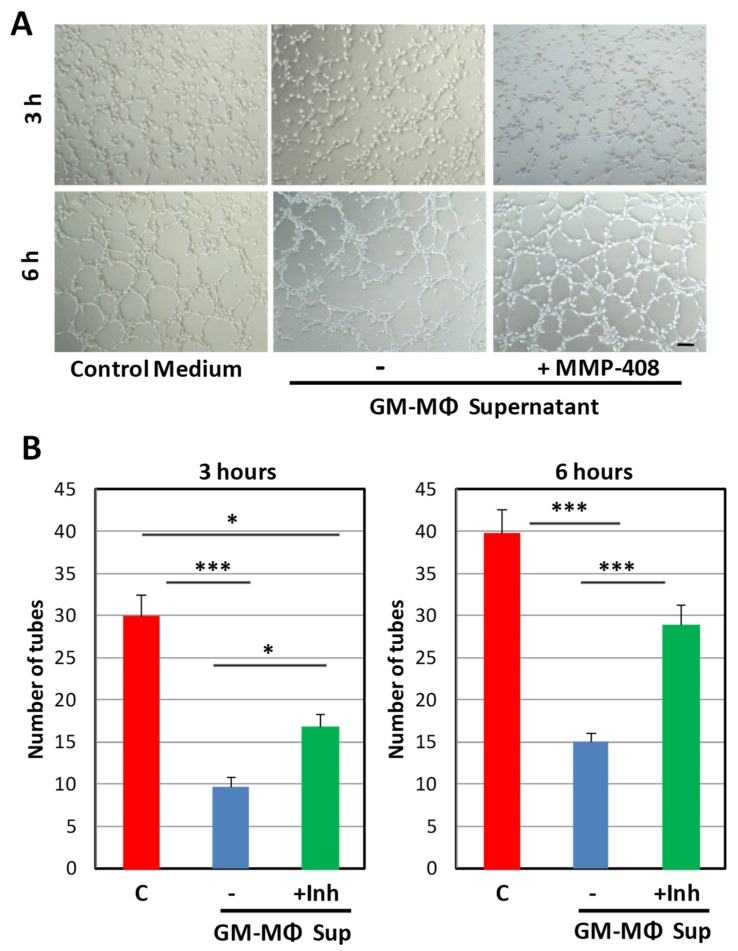
Effect of GM-MΦ supernatants on endothelial tubulogenesis in the presence of the MMP-12 inhibitor. HUVECs were incubated on Matrigel plates, as described in Figure 1, but in the absence (−) or presence (+Inh) of the MMP-12 inhibitor MMP-408, as indicated. Addition of MMP-408 significantly rescued the tubulogenesis inhibition induced by GM-MΦ supernatants. The cord network formation was visualized, and representative assays of more than three different experiments per condition (*n* = 6) are shown (**A**). Scale bar: 100 μm. The number of closed tubes in the network was quantified and represented by histograms (**B**). Statistical significance was calculated using one way ANOVA, and data are presented as mean ± SEM. (*p < 0.05; ***p < 0.001).

These results suggest that, among the MMP family, MMP-12 is a major component of the GM-MΦ secretome. Because MMP-12 has been associated with anti-angiogenic activity [30,31,32,33,34], we tested the possibility that MMP-12 was involved in the tubulogenesis inhibition induced by GM-MΦ supernatants. Supporting this hypothesis, we found that the MMP-12 inhibitor MMP-408 [56] significantly rescued the GM-MΦ supernatant-induced inhibition of endothelial tubulogenesis (Figure 3). These data suggest that MMP-12 is involved in the tubulogenesis inhibition induced by GM-MΦ supernatants.

### 2.2. Expression of Endoglin in Monocyte-Derived MΦ

**Figure 4:**
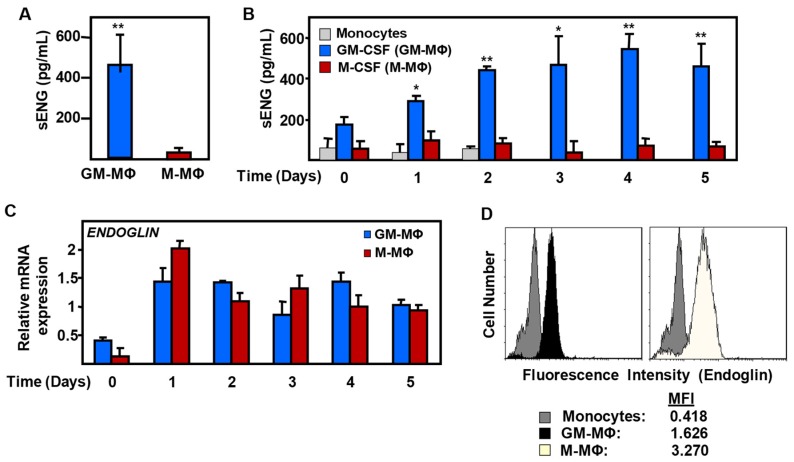
Endoglin expression in GM-MΦ and M-MΦ. Human monocytes were incubated with GM-CSF or M-CSF, resulting in GM-MΦ and M-MΦ, respectively. Soluble endoglin (sEng) levels were measured by ELISA in culture supernatants at the final stage of differentiation (**A**) or during a kinetic study (**B**). In panel **B**, the statistical significance with respect to the control sample (day 0) is indicated. (**C**) Comparative mRNA expression levels of endoglin along GM-MΦ and M-MΦ polarization, as determined by qRT-PCR. (**D**) Endoglin protein levels at the surface of GM-MΦ and M-MΦ were detected by immunofluorescence flow cytometry using a mouse monoclonal antibody directed against human endoglin. Values of mean fluorescence intensity (MFI) are indicated. Statistical analysis was calculated using the paired two-tailed Student *t* test (*n* = 3), and data are presented as mean ± SD. (*p < 0.05; **p < 0.01).

Because MMP-12 exerts its proteolytic activity on a variety of protein substrates, we searched for potential target proteins that could account for its anti-angiogenic function. Interestingly, we found that endoglin, a membrane protein present in MΦ, can be cleaved, at least, by MMP-14, generating a soluble form of endoglin with anti-angiogenic properties [40,51,53]. As MMP-14 is expressed at much lower levels than MMP-12 in GM-MΦ (Figure 2A) and no relevant differences were found between GM-MΦ and M-MΦ with respect to the transcript levels of MMP-14 (Figure 2B), we then focused on the potential targeting of endoglin by MMP-12. Human monocytes obtained from healthy donors and polarized in vitro showed different endoglin levels in their culture supernatant at the final stage of differentiation (Figure 4A). GM-CSF-polarized GM-MΦ presented levels of sEng up to 400 pg/mL, whereas M-CSF-polarized M-MΦ did not show detectable levels of sEng. 

A kinetic study showed that this effect was accumulative and measurable during the monocyte to MΦ transition, and the difference became significant 24 h after monocyte exposure to each cytokine (Figure 4B). By contrast, the levels of sEng released from non-stimulated monocytes were usually below detection range. The preferential presence of sEng in GM-MΦ supernatants agrees with the increased levels of sEng previously found in inflammatory-related pathologies [40,41,43,45,46,47]. Importantly, the differences in sEng levels between both MΦ subsets did not reflect parallel mRNA levels of endoglin (ENG), which were roughly similar between GM-MΦ and M-MΦ all along the differentiation process (Figure 4C). Moreover, significant differences were observed in the cell surface expression levels of endoglin in both MΦ subtypes (Figure 4D). Actually, there was a lower level of surface endoglin in GM-MΦ versus M-MΦ, a finding compatible with an active release of sEng in the GM-MΦ subset. Overall, these results are in line with the hypothesis that membrane endoglin is a target of GM-MΦ MMP-12.

### 2.3. Endoglin Shedding from GM-MΦ Cell Surface is Mediated by MMP-12

**Figure 5:**
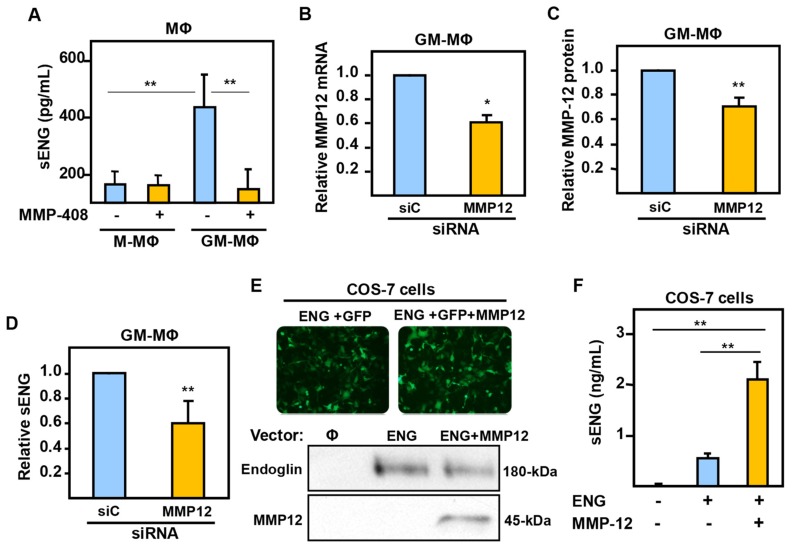
Expression of MΦ soluble endoglin induced by MMP-12. (**A**) Effect of the MMP-12 specific inhibitor MMP-408 on the release of soluble endoglin during in vitro macrophage polarization of human monocytes. (**B**–**D**) Effect of MMP-12 silencing in GM-MΦ. GM-MΦ were transfected with siRNA targeting MMP-12 or negative control (siC). After 48 h, MMP-12 mRNA (**B**) and protein (**C**) levels were determined by qRT-PCR and ELISA, respectively. Soluble endoglin levels were also measured in GM-MΦ culture supernatants (**D**). (**E**,**F**) Recombinant expression of MMP-12 releases soluble endoglin from membrane bound endoglin. COS-7 cells were co-transfected with endoglin (ENG), GFP, and MMP-12, as indicated. Transfection efficiency was visualized by fluorescence microscopy of GFP-expressing cells (**E**, upper panel) and by Western blot analysis (**E**, lower panel). A negative control with an empty vector (Φ) is included. Soluble endoglin levels were measured by ELISA in culture supernatants from transfected COS-7 cells. Statistical analysis was calculated using the paired two-tailed Student *t* test (*n* = 4), and data are presented as mean ± SD. (*p < 0.05; **p < 0.01).

The above hypothesis prompted us to analyze whether sEng was shed from the cell surface via the proteolytic activity of MMP-12 (Figure 5). The presence of the MMP-12-specific inhibitor MMP-408 during GM-CSF-driven polarization significantly reduced the concentration of sEng in GM-MΦ-conditioned medium, whereas the levels of sEng in M-MΦ supernatants remained low and unchanged (Figure 5A). In addition, transfection with siRNA directed to MMP-12 led to the down regulation of MMP-12 at the mRNA (Figure 5B) and protein (Figure 5C) level, and resulted in a significantly decreased secretion of sEng by GM-MΦ (Figure 5D). As another means to assess the ability of MMP-12 to shed endoglin from the cell surface, both MMP-12 and endoglin were over-expressed in COS-7 cells, which are devoid of endoglin and MMP-12 (Figure 5E). Co-transfection of both endoglin and MMP-12 resulted in a significant increase in the level of sEng compared to cells lacking MMP-12 expression (Figure 5F). These results suggest that the protease activity of MMP-12 is responsible for the presence of sEng shed by GM-MΦ.

### 2.4. MMP-12 Induces Soluble Endoglin Release in Endothelial Cells

**Figure 6:**
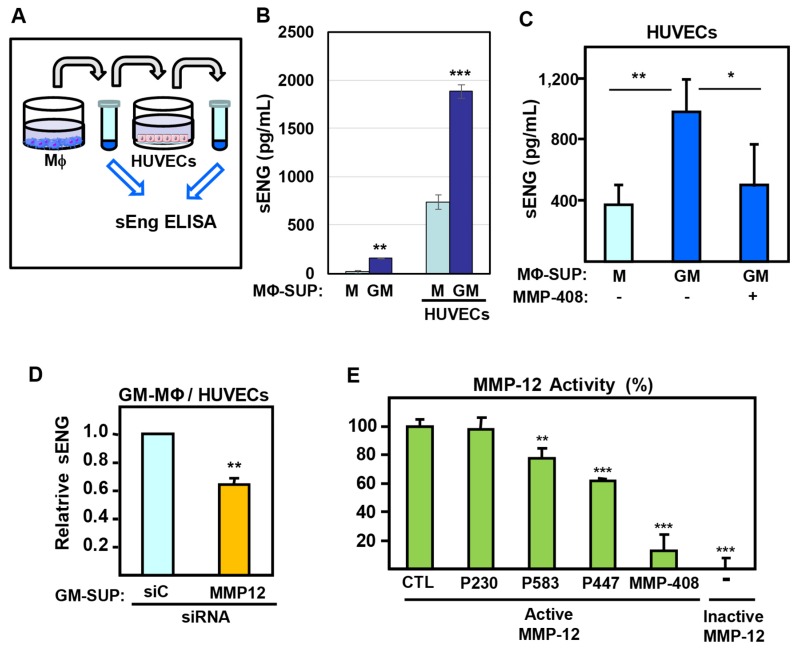
Secretion of endothelial soluble endoglin induced by MΦ supernatants. (**A**–**D**) As indicated in the scheme (**A**), culture supernatants (SUP) from GM-MΦ (GM) or M-MΦ (M) were added to monolayers of HUVECs. After 24 h incubation, the culture media were collected, and the sEng content was measured by ELISA in all samples. The values of sEng were calculated after subtracting the basal release of sEng present in HUVECs supernatants (**B**–**D**). (**B**) The levels of sEng from GM-MΦ and M-MΦ supernatants (left two bars) are compared to those after incubation with HUVECs (right two bars). (**C**) Soluble endoglin levels from GM-MΦ and M-MΦ supernatants after incubation with HUVECs in the presence or absence of MMP-408. (**D**) Soluble endoglin levels from supernatants of GM-MΦ, previously transfected with siRNA-MMP-12 or -control (siC), after incubation with HUVECs. (**E**) Effect of endoglin peptides (P230, P583, P447) and the specific inhibitor MMP-408 on the proteolytic activity of recombinant human MMP-12. Control samples with vehicle (CTL), the GL-less peptide P230, as well as an inactive MMP-12, are included. The MMP-12 activity was measured using the fluorogenic substrate Mca-PLGL-Dpa-ARNH2, and the statistical significance respect to the control sample (CTL) is indicated. Statistical analysis (*n* = 3) was calculated using the ANOVA test, and data are presented as mean ± SD. (*p < 0.05; **p < 0.01; ***p < 0.001).

Next, we sought to determine whether GM-MΦ-derived MMP-12 can also act on endothelial cells, which constitute the major source of endoglin. To that end, primary cultures of HUVECs were exposed to GM-MΦ- or M-MΦ-conditioned media (Figure 6A). As shown in Figure 6B, the GM-MΦ-conditioned medium triggered a significantly higher level of HUVEC-derived sEng, as compared to the M-MΦ medium. Of note, the levels of sEng in MΦ-supernatants were much lower than those obtained after incubation with HUVECs (Figure 6B). 

The involvement of MMP-12 in the endothelial shedding of sEng was then assessed. First, the levels of endothelial sEng induced by the GM-MΦ-conditioned medium were inhibited in the presence of the MMP-12 inhibitor MMP-408 (Figure 6C). Next, a significantly decreased expression of sEng was detected in HUVECs exposed to supernatants from GM-MΦ transfected with siRNA-MMP-12 (Figure 6D). Furthermore, the ability of MMP-12 to target the endoglin sequence was evaluated using an in vitro assay with recombinant MMP-12 and an MMP-12-sensitive fluorogenic peptide. As expected, the MMP-12 specific inhibitor MMP-408 [56] decreased the proteolytic activity of MMP-12 by 80% (Figure 6E). Interestingly, a significant inhibition of MMP-12 activity on the fluorogenic peptide was also observed in the presence of endoglin-specific peptides P583 and P447, where both of them include the consensus Gly-Leu (GL) recognition sequence for MMPs, which is in agreement with their inhibitory effect against MMP-14 activity on endothelial endoglin [57]. As a negative control, the GL-less peptide P230 had no effect on the proteolytic activity of MMP-12. Overall, these data suggest that MMP-12 is capable of proteolytically targets membrane-bound endoglin in endothelial cells.

### 2.5. MMP-12 Induces Soluble Endoglin Release in an in Vivo Mouse Model of Inflammation

**Figure 7:**
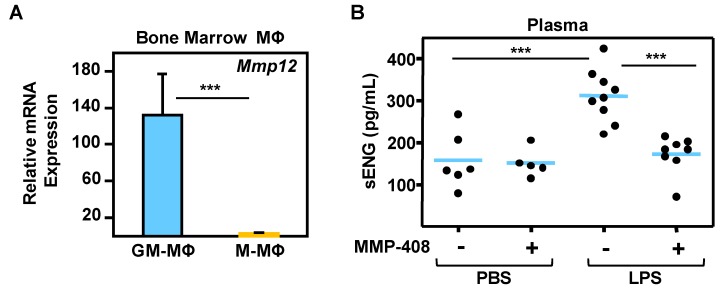
MMP-12-dependent release of soluble endoglin in mice. (**A**) Transcript expression levels of Mmp-12 in murine bone marrow-derived MΦ incubated with GM-CSF (GM-MΦ) or M-CSF (M-MΦ), as determined by qRT-PCR. Statistical significance of data (*n* = 4) was calculated using a paired, two-tailed Student *t* test, and data are presented as mean ± SD. (**B**) Soluble endoglin levels in lipopolysaccharide (LPS)-treated animals. Mice were injected (i.p.) with LPS and orally gavaged with MMP-408 or vehicle (PBS). Plasma samples were extracted, and soluble endoglin was measured using an ELISA kit. A point cloud representation is shown where blue horizontal bars indicate the median values of each condition. Statistical significance was calculated (*n* ≥ 5) using the ANOVA test, and data are presented as mean ± SD.

As a means to address the physiological relevance of the MMP-12-dependent sEng release, we initially analyzed the expression of MMP-12 in murine bone marrow-derived GM-MΦ and M-MΦ. GM-CSF-driven polarization resulted in higher levels of Mmp-12 mRNA than in M-CSF-treated macrophages (Figure 7A), which is in agreement with findings from human polarized MΦ (Figure 2). Based on this result, we undertook the analysis of the levels of circulating sEng in response to lipopolysaccharide (LPS), a powerful stimulus of acute inflammatory responses. The i.p. injection of LPS and the non-lethal associated inflammatory response resulted in a significant increase of sEng levels in the plasma after 48 h (Figure 7B), indicating that the release of sEng is also a marker for the LPS-induced in vivo inflammatory response. Importantly, the LPS-induced increase in plasma sEng was significantly abolished by the MMP-12 inhibitor MMP-408, whose only presence had no effect on the basal levels of sEng in plasma (Figure 7B). Together, these results suggest that MMP-12 is also involved in the release of sEng in an in vivo inflammatory mouse model.

## 3. Discussion

**Figure 8:**
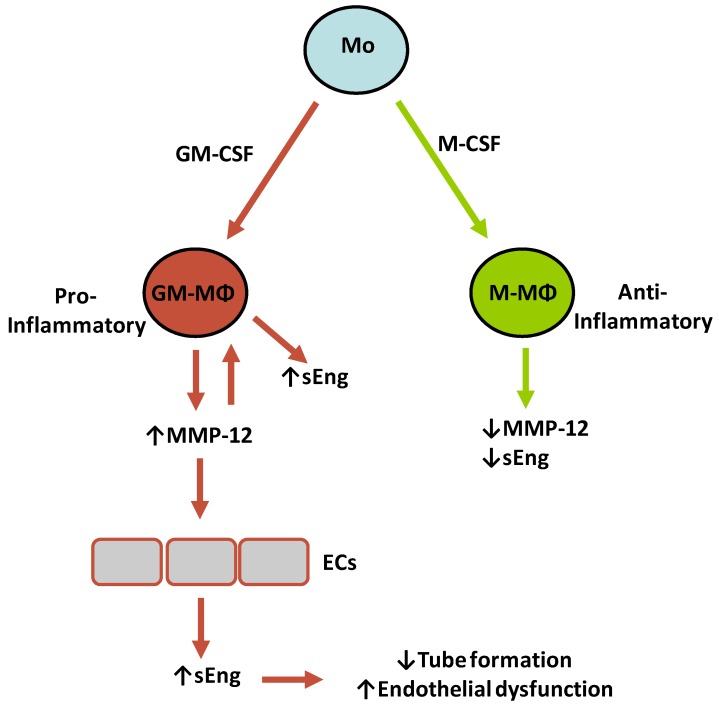
Hypothetical model for MMP-12 in sEng secretion and endothelial function. Circulating monocytes (Mo) can be polarized in the presence of GM-CSF or M-CSF, leading to pro-inflammatory GM-MΦ or anti-inflammatory M-MΦ, respectively. GM-MΦ, but not M-MΦ, secrete high levels of MMP-12, which in turn triggers the release of sEng in GM-MΦ and endothelial cells (ECs) by proteolytically cleaving membrane-bound endoglin. The effects of sEng include inhibition of tube formation and a pro-inflammatory stimulus on ECs that may lead to endothelial dysfunction.

Macrophages regulate a variety of processes involving vascular inflammation. There is a wide range of vascular inflammatory diseases, including atherosclerosis, hypertension, vasculitides aortic aneurism, or rare vascular syndromes [3,7,58]. Together, these are widespread conditions that affect a large population worldwide, sometimes leading to fast and irreversible organ failure. Polarization and accumulation of MΦ upon chronic pro-inflammatory signals may result in extensive vascular damage, adverse repair, and worsened clinical outcomes [59]. Therefore, targeting the inflammatory MΦ activation may lead to an early resolution of inflammation with potential therapeutic benefits. Unfortunately, how MΦ regulate the inflammatory responses in the vasculature is poorly understood. In this context, elucidating novel mechanisms of MΦ polarization may lead to a better understanding of their roles in vascular inflammation. In this manuscript, we report a new link between pro-inflammatory GM-MΦ and endothelial cells, by which GM-MΦ-derived MMP-12 targets membrane bound endothelial endoglin, leading to the release of sEng, a soluble protein with pro-inflammatory properties and the potential to promote endothelial dysfunction in combination with hypercholesterolemia [55]. Our results suggest that there is a direct and synergic effect of both MMP-12 and sEng in the context of the GM-MΦ/pro-inflammatory macrophage response (Figure 8). Supporting this view, it has been reported that in inflammation-associated pathologies in which MMP-12 levels are increased [26,29,60], there is also an augmented level of sEng [44,61,62,63]. Furthermore, we found that GM-MΦ supernatants show an MMP-12-dependent inhibitory activity on endothelial tubulogenesis, which is in agreement with the reported anti-angiogenic effect of both sEng [40,47,53] and MMP-12 [30,31,32,33]. 

These data suggest that both soluble proteins act in concert, contributing to the progression of the GM-MΦ response in both physiological and pathological processes. In this line, several lines of evidence support the functional relationship between MΦ and endoglin. Thus, MΦ endoglin knockout mice show a predisposition to develop spontaneous infections by opportunistic bacteria, increased survival following LPS-induced peritonitis, and impaired phagocytic activity in endoglin deficient peritoneal MΦ [64]. Interestingly, this altered function of endoglin-deficient MΦ could help to explain the higher rate of infectious diseases seen in patients with HHT1, a vascular disease caused by heterozygous mutations in the endoglin gene [65,66]. This is also in agreement with the impaired resolution of inflammation reported in an endoglin heterozygous HHT1 mouse model of chronic colitis [67]. In addition, endothelial endoglin can regulate integrin-dependent monocyte adhesion and extravasation upon inflammatory stimuli, and these processes can also be modulated by sEng [13]. As the inflammatory infiltrate of MΦ plays a critical role in vascular remodeling [58] and such infiltrate has been reported in vascular lesions of HHT1, a pathogenic role for the impaired monocyte/MΦ infiltration in this disease has been postulated [38,68,69]. 

Macrophages are critical components in inflammatory diseases that occur within and around blood vessels and lead to severe vascular damage and tissue ischemia. Integrating the new findings reported here can unravel novel therapeutic targets through better understanding of the pro-inflammatory crosstalk between MΦ and the endothelium. In the context of chronic inflammation in the vascular disease, studies on MΦ and endothelial biology are key to harnessing inflammation.

## 4. Materials and Methods 

### 4.1. Macrophage Culture and Differentiation

All cells were incubated routinely at 37 °C in a humidified atmosphere with 5% CO_2_. This study was approved by the Centro de Investigaciones Biológicas Ethics Committee, and all experiments were carried out in accordance with institutional guidelines and regulations. Blood-derived buffy coats of normal donors were anonymously provided by the Comunidad Autonoma de Madrid Blood Bank. Human peripheral blood mononuclear cells (PBMCs) were isolated from buffy coats over a Lymphoprep (Nycomed Pharma, Oslo, Norway) gradient according to standard procedures. Monocytes were purified from PBMCs by magnetic cell sorting using CD14 microbeads (Miltenyi Biotech, Bergisch Gladbach, Germany). Monocytes (>95% CD14+ cells) were cultured at 0.5 × 10^6^ cells/mL for 4–7 days in RPMI media supplemented with 10% fetal bovine serum (FBS, Gibco; complete medium), containing 1000 U/mL GM-CSF or 10 ng/mL M-CSF (ImmunoTools GmbH, Friesoythe, Germany) to generate GM-MΦ and M-MΦ monocyte-derived macrophages, respectively. Cytokines were added every two days. When necessary, the MMP-12 specific inhibitor MMP-408 (10 µg/mL) was added to the culture media.

### 4.2. Endothelial Cell Culture

Human umbilical vein-derived endothelial cells (HUVECs) were purchased from Lonza (Basel, Switzerland) and used at early passages [47]. HUVECs were grown on 0.2% gelatin (Sigma-Aldrich, St. Louis, MO, USA) pre-coated plates in endothelial basal medium (EBM2) supplemented with 10% heat-inactivated FBS and EGM2 SingleQuots (EBM2/EGM2 medium; Lonza, Basel, Switzerland) unless otherwise noted. When indicated, HUVECs were cultured in the presence of MΦ conditioned media. To this end, primary cultures of HUVECs were grown in EBM2/EGM2 medium until 90% confluency. After removing the media, cells were washed twice with PBS. Supernatants of previously polarized GM-MΦ or M-MΦ (6 days) were centrifuged, added to the HUVEC monolayers, and then incubated for 24 h. The resulting culture supernatants were centrifuged and stored for further analysis.

### 4.3. Tube Formation and wound Healing Assays

In vitro tubulogenesis and wound healing assays were performed as previously described [47]. Briefly, for tube formation assays, HUVECs were seeded on 24-well plates previously covered with 120 μL standard Matrigel (BD Bioscience, San Jose, CA, USA) diluted 1:1 in serum-free RPMI medium or MΦ supernatants for 3 h and 6 h, as indicated. Images were taken with an Olympus digital camera, and quantification of closed tubes (tubular structures/microscopic field) was performed using Fiji-ImageJ software. In vitro scratch wounds were created by scraping confluent HUVEC monolayers in 24-well plates with a sterile pipette tip. Fresh EBM2 medium supplemented with 2% FBS and EGM2 SingleQuots diluted 1:1 with MΦ supernatants was added, and samples were incubated for up to 10 h. Endothelial cell migration into the denuded area was monitored at different times and recorded at 8 h post-wound. The ImageJ program [70] was used to quantify the wound healing process.

### 4.4. Quantitative RT-PCR 

Total RNA was extracted using an RNeasy kit (Qiagen, Hilden, Germany), retrotranscribed, and amplified in triplicates. Oligonucleotides for selected human and mouse genes (*ENG*, *MMP-1*, *MMP-2*, *MMP-7*, *MMP-9*, *MMP-12*, *MMP-14*, *MMP-15*, *MMP-17*, *MMP-19*, *MMP-23B*, *MMP-25*, *MMP-28*, *Mmp-12*) were designed according to the Universal ProbeLibrary system (Roche Diagnostics, Mannheim, Germany) for quantitative real-time PCR (qRT-PCR). Results were expressed relative to the expression level of glyceraldehyde 3-phosphate dehydrogenase (GAPDH). For siRNA-transfected MΦ, total RNA was extracted using the NucleoSpin RNA/Protein kit (Macherey-Nagel, Düren, Germany), retrotranscribed, and amplified using the Universal Human Probe Library (Roche Diagnostics, Mannheim, Germany). Assays were made in triplicate, and results were normalized according to the expression levels of TATA-binding protein (TBP) and hypoxanthine phosphoribosyltransferase 1 (HPTR1) genes.

### 4.5. Immunodetection Assays

Western blot analysis was performed as described previously [71]. Immunodetection was carried out by probing the membrane with a mouse monoclonal antibody (mAb) against human endoglin (P4A4; Developmental Studies Hybridoma Bank, University of Iowa, Iowa City, IA, USA) or the V5 artificial epitope (Sigma-Aldrich, Saint Louis, MO, USA) overnight at 4 °C, followed by incubation with the proper horseradish peroxidase-conjugated secondary antibody. Culture supernatants from HUVECs, GM-MΦ, M-MΦ, or COS-7-lipofected cells were collected and centrifuged at 11,000× *g* for 10 min to remove cellular debris. The presence of human sEng or MMP-12 in culture supernatants was analyzed using the Endoglin/CD105 Quantikine (DNDG00, R&D Systems, Minneapolis, MN, USA) or MMP-12 (LS-F24617, LSBio, Seattle, WA, USA) ELISA kit, respectively, according to the manufacturers’ instructions. For immunofluorescence flow cytometry analysis, cells were collected, centrifuged, and washed twice with PBS at 4 °C. After blocking with 2% human AB^+^ serum in PBS for 30 min, cells were incubated with the mouse mAb anti-endoglin (P4A4) or a nonspecific (isotype-matched) antibody (negative control), followed by incubation with Alexa Fluor 488 goat anti-mouse antibodies (Invitrogen, Waltham, MA, USA). The samples were analyzed in an EPICS Coulter XL flow cytometer.

### 4.6. Recombinant MMP-12 Activity

Recombinant human MMP-12 (R&D Systems, Minneapolis, MN, USA) activity was determined with a quenched fluorogenic peptide Mca-PLGL-Dpa-AR-NH2 (R&D Systems, Minneapolis, MN, USA), as previously described [57]. Briefly, MMP-12 was activated by incubation with an assay buffer (50 mM Tris, 10 mM CaCl_2_, 150 mM NaCl, 0.05% Brij-35; pH 7.5) at 37 °C for 30 h. The fluorescence intensity was measured with a Varioskan flash spectral scanning multimode reader (Thermo Scientific, Waltham, MA, USA). When necessary, a mixture of 0.4 ng/mL of activated rhMMP-12 with 6 µM of fluorogenic peptide was incubated with 30 µM endoglin peptides or the MMP-12 inhibitor MMP-408. Endoglin-related peptides P583 (TSKGLVLP), P447 (SLSFQLGLYL), and P230 (GPRTVTVK) with N-terminal acetylation and C-terminal amidation [57] were synthesized and purified to >90% purity (Biomedal SL, Sevilla, Spain).

### 4.7. Generation of the MMP-12 Expression Vector

To generate the recombinant vector, GM-MΦ mRNA was retrotranscribed using the iScript complementary DNA synthesis kit (Bio-Rad, Hercules, CA, USA), followed by amplification by PCR using a high fidelity Taq polymerase (5 Prime) with specific primers for human MMP-12 (Fw: 5’-ATG-CGG-TAC-CAT-GGG-GAA-GTT-TCT-TCTAAT-A-3’; and Rv: 5’-ATG-CCT-CGA-GCT-AAC-AAC-CAAACC-AGC-TAT-TGC-TTT-3’). “TA cloning” was performed in the pcDNA3.1/V5-His TOPO vector according to manufacturer’s instructions (TA Expression Kit, Invitrogen, Waltham, MA, USA). Reliability of the resulting construct (pcDNA3.1-MMP-12) was checked by enzymatic digestion (BamHI-XhoI) and sequencing (Secugen, Madrid, Spain).

### 4.8. Transient Transfection Assays

For transient transfection studies the monkey kidney, the COS-7 cell line was grown in Dulbecco’s modified Eagle’s medium supplemented with 10% FBS. COS-7 cells were transiently transfected with expression vectors pcDNA3.1-MMP-12 and pDisplay-ENG [72], encoding human MMP-12 and endoglin, respectively. Control transfections with pDisplay empty vector (Invitrogen) or GFP vector (Lonza, Basel, Switzerland) were also included. Transfection experiments were performed with Lipofectamine LTX (Invitrogen) according to the manufacturer’s instructions. After 48 h, culture supernatants were collected and the cells were lysed in lysis buffer (10 mM Tris-HCl pH 8.0, 150 mM NaCl, 1% NP-40, and a cocktail of protease and phosphatase inhibitors) for later ELISA, western blot, and zymography analyses. For MMP-12 silencing, GM-MΦ (1 × 10^6^ cells/mL) were transfected with a pool of MMP-12-specific small interfering RNA (s92, si93, and si94; 50 nM each; Sigma) using Hiperfect (#301705, Qiagen, Hilden, Germany). As a negative control, cells were transfected with a nonspecific small interfering RNA control (siControl; Life Technologies, Carlsbad, CA, USA). After transfection, cells were cultured for the indicated times in RPMI 1640 supplemented with 10% FBS and subjected to mRNA and protein analysis. 

### 4.9. Casein Zymography

SDS-polyacrilamide gels (12%) were co-polymerized with 0.5 mg/mL bovine β-casein (Sigma). After pre-running the gels, samples (cell culture supernatants) in Laemmli buffer 1× without reducing agents were loaded without boiling and electrophoresed at 100 V. SDS was removed after the run by washing the gel twice with 50 mM Tris-HCl (pH 7.5) and 2.5% Triton X-100 for 30 min and twice more for 10 min with 50 mM Tris-HCl (pH 7.5). The gel was incubated overnight at 37 °C with 50 mM Tris-HCl (pH 7.5), 0.15 M NaCl, 5 mM CaCl_2_, 2.5% Triton X-100, and 0.02% NaN_3_. The staining was performed for 1 h at room temperature with 0.5% Coomassie Brilliant Blue R-250 in 10% acetic acid, followed by destaining with 10% acetic acid until digestion bands were visible.

### 4.10. In Vivo Inflammatory Response and Inhibition of MMP-12 in a Mouse Model

Ten-week-old mice were injected (i.p.) with 1 mg/kg body weight of lipopolysaccharide (LPS; from E. coli O111:B:4, Sigma-Aldrich, St. Louis, MO, USA) or PBS as a control condition. Three hours before LPS injection, mice were orally gavaged with 150–200 µL of 0.5% methylcellulose, 2% Tween-80 (vehicle), or MMP-408 (30 mg/kg body weight; Merck-Millipore, Burlington, MA, USA). Vehicle or MMP-408 gavage was repeated 24 h after i.p. injection with LPS. All animals were handled in strict accordance with good animal practice as defined by the national animal welfare bodies (RD 1201/2005 BOE #252). The experimental design and all animal work were approved by our institutional Ethical Committee for animal experiments. Mice were deeply anesthetized, and blood samples were obtained by puncture of posterior vena cava. The blood was centrifuged for 15 min at 1000*g* to collect serum samples. Levels of mouse sEng in serum were determined by DuoSet^®^ ELISA Mouse Endoglin/CD105 Immunoassay (R&D Systems, Minneapolis, MN, USA).

### 4.11. Statistical Analysis

All the experiments were performed with ≥3 replicates in each condition and repeated, at least, three times with similar results. Statistical analyses were performed on GraphPad Prism 8 for Windows (La Jolla, CA, USA). Statistical significance was calculated using the paired two-tailed Student *t* test or by ANOVA for multiple comparison and Tukey’s test. Statistical data are presented either as mean ± standard deviation (SD) or as mean ± standard error of the mean (SEM), as indicated. A value of *p < 0.05 was considered significant; **p < 0.01; ***p < 0.001.

## Abbreviations

FBS	Fetal Bovine Serum
GM-CSF	Granulocyte-Macrophage Colony-Stimulating Factor
GM-MΦ	GM-CSF monocyte-derived macrophage
HHT	Hereditary Hemorrhagic Telangiectasia
HUVECs	Human Umbilical Vein Endothelial Cells
MΦ	Macrophages
M-CSF	Macrophage Colony Stimulating Factor 1
M-MΦ	M-CSF monocyte-derived macrophage
MMP	Matrix Metalloproteinase
PBMCs	Peripheral Blood Mononuclear Cells
sEng	soluble Endoglin
SDS	Sodium dodecyl sulfate
qRT-PCR	quantitative Real-Time PCR
